# Dietary Supplementation with *Rhodotorula mucilaginosa* Enhances Resistance to *Aeromonas veronii* Infection in Red Claw Crayfish (*Cherax quadricarinatus*)

**DOI:** 10.3390/ani15131912

**Published:** 2025-06-28

**Authors:** Qin Zhang, Liuqing Meng, Haoliang Lu, Luoqing Li, Qinghui Zeng, Dapeng Wang, Rui Wang, Tong Tong, Yongqiang Liu, Huizan Yang

**Affiliations:** 1 Guangxi Key Laboratory for Polysaccharide Materials and Modifications, School of Marine Sciences and Biotechnology, Guangxi Marine Microbial Resources Industrialization Engineering Technology Research Center, Guangxi Minzu University, 158 University Road, Nanning 530008, China; zhangqin@gxmzu.edu.cn (Q.Z.); mengliuqing@stu.gxmzu.edu.cn (L.M.); luhaoliang@stu.gxmzu.edu.cn (H.L.); liluoqing@stu.gxmzu.edu.cn (L.L.); zengqinghui@stu.gxmzu.edu.cn (Q.Z.); tongtong@gxmzu.edu.cn (T.T.); 2 Guangxi Key Laboratory for Aquatic Genetic Breeding and Healthy Aquaculture, Guangxi Academy of Fishery Sciences, 8 Qingshan Road, Nanning 530021, China; oucwdp@163.com (D.W.); raywongxx@163.com (R.W.)

**Keywords:** *Cherax quadricarinatus*, *Rhodotorula mucilaginosa*, antioxidant, immune, *Aeromonas veronii*

## Abstract

The objective of this study was to evaluate the effects of dietary supplementation with different levels of *Rhodotorula mucilaginosa* (0.0 g/kg, 0.1 g/kg, 1.0 g/kg, and 10.0 g/kg) on resistance to *Aeromonas veronii* infection in red claw crayfish (*Cherax quadricarinatus*) (initial body weight of 0.13 ± 0.06 g). The investigation combined a 56-day feeding trial with a subsequent 7-day infection challenge to assess cumulative mortality, immune and antioxidant enzyme activities, and the relative expression of immune-related genes. During the *A. veronii* infection test, the cumulative mortalities for the 0.1 g/kg, 1.0 g/kg, and 10.0 g/kg groups were 44.44%, 38.89%, and 38.89%, respectively, all significantly lower (*p* < 0.05) than that of the control group (58.33%). Compared with the control group, after infection with *A. veronii*, the activities of acid phosphatase, alkaline phosphatase, catalase, and superoxide dismutase in the hepatopancreas and alkaline phosphatase, lysozyme in the hemolymph of red claw crayfish in the 1.0 g/kg group significantly increased (*p* < 0.05). The activities of aspartate aminotransferase and alanine aminotransferase in the hemolymph of red claw crayfish in the 1.0 g/kg group significantly decreased (*p* < 0.05). The relative expression levels of serine protease inhibitor, crustacean hyperglycemic hormone, anti-lipopolysaccharide factor, and superoxide dismutase genes in the hepatopancreas of red claw crayfish in the 1.0 g/kg group were significantly upregulated (*p* < 0.05). In conclusion, *R. mucilaginosa* could significantly improve the antibacterial ability of red claw crayfish against *A. veronii*. In this experimental context, the ideal addition level of *R. mucilaginosa* is determined to be 1.0 g/kg.

## Simple Summary

In aquaculture, red claw crayfish (*Cherax quadricarinatus*) often die from *Aeromonas veronii* infection, leading to economic losses. This study aimed to find out whether adding different amounts of *Rhodotorula mucilaginosa* (0 g/kg, 0.1 g/kg, 1.0 g/kg, and 10.0 g/kg) to their diet could help them fight off *A. veronii* infections. The experiment first involved feeding crayfish these different diets for 56 days, followed by exposing them to *A. veronii* for 7 days. The results show that crayfish fed diets with 0.1 g/kg, 1.0 g/kg, and 10.0 g/kg of *R. mucilaginosa* had lower death rates. With this amount, the crayfish showed increased activity of several enzymes important for the immune system. Some key immune-related genes also became more active, while enzymes indicating liver damage decreased. In conclusion, *R. mucilaginosa* can significantly boost crayfish’s ability to resist *A. veronii*. Based on this experiment, adding 1.0 g/kg of *R. mucilaginosa* to the feed is the optimal choice.

## 1. Introduction

The red claw crayfish (*Cherax quadricarinatus*) stands as one of the most globally prominent freshwater crustacean species in aquaculture, having secured its indispensable industrial status through a combination of advantageous traits. These include rapid growth rates, succulent flesh quality, and remarkable environmental resilience [1]. However, the progressive intensification of stocking densities and expansion of production scales have rendered red claw crayfish increasingly susceptible to diverse pathogenic incursions, thereby exacerbating the risks of disease transmission [2]. Notably, within high-density recirculating aquaculture systems, *Aeromonas* infections exhibit profoundly efficient transmission dynamics [3]. *Aeromonas veronii*, a Gram-negative facultative anaerobic bacterium distinguished by its remarkable environmental tenacity and disseminating capability, demonstrates robust proliferation under intensive aquacultural conditions, and this pathogen inflicts severe pathological sequelae through hepatopancreatic dysfunction and intestinal epithelial disruption in red claw crayfish [4].

*A. veronii* functions not only as a primary aquatic pathogen but also as an emerging zoonotic agent capable of inducing gastroenteritis and septicemia in humans, thereby posing dual menaces to public health and sustainable aquacultural development [5,6]. While conventional antibiotic therapies afford immediate disease control, their prolonged utilization harbors risks of fostering antimicrobial resistance and contaminating aquatic products, significantly impeding industry sustainability [7]. This scenario has thrust probiotics into a position of prominence as environmentally benign alternatives [8]. Empirical evidence demonstrates that probiotics augment red claw crayfish growth performance and disease resistance via multifaceted mechanisms that include pathogen growth inhibition, nutrient assimilation enhancement, gut microbiota modulation, and immune system potentiation [8].

*Rhodotorula mucilaginosa*, a burgeoning probiotic candidate, has attracted substantial interest owing to its rich repertoire of bioactive constituents that include carotenoids, polyunsaturated fatty acids, and functional enzymes [9]. *R. mucilaginosa* synthesized carotenoids, such as β-carotene and torularhodin, exhibit potent antioxidant properties and effectively scavenge reactive oxygen species to mitigate oxidative stress [10]. Additionally, its polyunsaturated fatty acids possess immunomodulatory attributes that enhance anti-inflammatory responses [11]. Study has confirmed that dietary supplementation with the *R. mucilaginosa* strain in Pacific white shrimp (*Penaeus vannamei*) significantly elevates growth parameters, immune indices, and resistance against *Vibrio parahaemolyticus* [12]. Notwithstanding these advancements, substantial knowledge gaps persist regarding the optimal dosage, mechanistic pathways, and context-dependent efficacy of *R. mucilaginosa* against *A. veronii* in red claw crayfish aquaculture systems. This study aims to systematically characterize the protective effects of *R. mucilaginosa* against *A. veronii* infection in red claw crayfish and thereby furnish a scientific basis for sustainable cultivation protocols.

## 2. Materials and Methods

### 2.1. Experimental Diets

The freeze-dried powder of *R. mucilaginosa*, supplied by Guangzhou Xinhaili Biotechnology Co., Ltd. (Guangzhou, China), contained over 1 × 10^10^ cells per gram. The nutritional profile of *R. mucilaginosa* (based on the wet weight) was as follows: 81.17% moisture, 9.27% crude protein, 4.45% crude lipid, 4.2% total triglycerides, 1.3% β-glucan, 1.4 mg/kg β-carotene, 1.0 mg/kg astaxanthin, and 172 mg/kg vitamin E. *R. mucilaginosa* was stored at −20 °C in a refrigerator.

The safety assessment of *R. mucilaginosa* was conducted following the methodology of Kang et al. [13]. Healthy juvenile crayfish (purchased from the South Propagation Base of Guangxi Academy of Fishery Sciences, Nanning, China; initial body weight: 1.10 ± 0.09 g and initial body length: 3.08 ± 0.12 cm) were randomly allocated into two groups (treatment and control), each with three replicates. A total of six aquaculture tanks (0.6 m × 0.6 m × 0.6 m) were used, with ten crayfish per tank. Water parameters were maintained as follows: water temperature of 26–28 °C, dissolved oxygen of 4.7–6.2 mg/L, ammonia nitrogen of 0–0.25 ppm, nitrite of 0–0.01 ppm, nitrate of 0–10 ppm, pH 7.6–7.8, and a natural photoperiod (10 h light:14 h dark). In the treatment group, *R. mucilaginosa* was added to maintain a concentration of 1 × 10^8^ CFU/mL in the aquaculture water, whereas the control group received no bacterial supplementation. Daily management included renewing one-third of the water volume, removing bottom feces, replenishing 1 × 10^8^ CFU/mL *R*. *mucilaginosa* (treatment group), and providing a basal diet. Juvenile crayfish were observed for 7 days to monitor feeding behavior and survival, with daily records of survival counts. By the end of the cultivation period, all red claw crayfish exhibited optimal growth and vitality, with no significant reduction in feeding activity. Both groups achieved a 100% survival rate.

All feed materials were of animal food grade and manufactured by Guangdong Hengxing Feed Industry Co., Ltd. (Zhanjiang, China). Fishmeal (Peruvian type, protein ≥ 65%) and soybean meal (protein ≥ 46%) were used as primary protein sources. Based on previous research [14] and safety assessment results (1 × 10^8^ CFU/mL), experimental diets were formulated with varying concentrations of *R. mucilaginosa*. To prepare the feed with 35.20% crude protein and 7.84% crude lipids, specific amounts of freeze-dried *R. mucilaginosa* powder were incorporated into the basal feed and mixed thoroughly to achieve final concentrations of 0 g/kg, 0.1 g/kg, 1.0 g/kg, and 10.0 g/kg. First, the basal feed was ground to a 60-mesh particle size using a hammer mill. Subsequently, the designated concentrations of *R. mucilaginosa* were added to the ground basal feed, and all mixtures were homogenized for 15 min in a drum mixer. Sterile distilled water was then added at a 40% (*w*/*w*) ratio to form a dough-like consistency. The moistened mixtures were extruded through a pellet mill to produce pellets with diameters of 1.00–1.50 mm. The feed pellets were air-dried at 30 °C until their moisture content dropped below 10 g/100 g. The dried pellets were then sealed in airtight bags and stored at −20 °C for later use. Fresh feed was prepared weekly. The specific ingredients of the experimental diets are listed in Table 1.

The viable count of *R. mucilaginosa* in feed was evaluated by plate counting [15]. A stock solution was prepared by vortexing 1 g of feed sample in 9 mL sterile normal saline. Serial dilutions (10^3^–10^8^) were made from the stock solution. For each dilution, 0.1 mL was plated onto sterile nutrient agar plates in triplicate. Plates were incubated anaerobically at 35 °C for 48 h. After incubation, colonies were counted, and random colonies were selected for identification to confirm *R. mucilaginosa* purity. The number of *R. mucilaginosa* colonies per plate was calculated. Post-counting, additional colonies were randomly chosen for strain identification and isolation. The calculation formula is as follows:

$$The\;number\;of\;viable\;R.\;mucilaginosa=B\times \frac{C}{10}\times f$$

In the above formula, B represents the total number of colonies on nutrient agar plates, C denotes the number of *R. mucilaginosa* colonies identified from 10 randomly selected colonies, and f signifies the dilution factor.

According to the NA plate counting results, no viable *R. mucilaginosa* was detected in the control group diet. Conversely, the viable counts of *R. mucilaginosa* in the experimental diets supplemented with 0.1 g/kg, 1.0 g/kg, and 10.0 g/kg were 0.89 × 10^6^ CFU/g, 0.87 × 10^7^ CFU/g, and 0.92 × 10^8^ CFU/g, respectively.

### 2.2. Experimental Animals and Feeding Management

The red claw crayfish utilized in the experiment were obtained from the South Propagation Base of the Guangxi Academy of Fishery Sciences, located in Nanning, China. The initial body weight was 0.13 ± 0.06 g, and the initial body length was 0.58 ± 0.02 cm. Prior to the culture experiment, the crayfish were acclimated in the laboratory’s water recirculation system for 7 days. After acclimation, juveniles with a uniform body morphology, no limb abnormalities, a healthy appearance, and strong vitality, as well as in the intermolt stage, were selected for the experiment.

The experiment was designed with the following four groups based on dietary *R. mucilaginosa* concentrations: a control group (0 g/kg), a low-dose group (0.1 g/kg), a medium-dose group (1.0 g/kg), and a high-dose group (10.0 g/kg). Each group had three replicates, resulting in a total of 12 aquaculture tanks. Each tank contained 50 crayfish, yielding a total of 600 crayfish across all tanks. The experimental rearing tanks measured 1 m × 2 m × 1 m (length × width × height) with a water depth of 0.70 m. Each tank was equipped with 16 PVC pipes (diameter: 0.75 m and length: 0.40 m) as shelters. Aeration stones provided continuous aeration, while aquatic plants were introduced to simulate a natural ecological environment, thereby reducing stress responses and increasing dissolved oxygen levels for the crayfish. Environmental conditions were strictly regulated, as follows: water temperature of 26–28 °C, dissolved oxygen of 4.7–6.2 mg/L, ammonia nitrogen of 0–0.25 ppm, nitrite of 0–0.01 ppm, nitrate of 0–10 ppm, pH 7.6–7.8, and a natural photoperiod (10 h light:14 h dark).

During the formal rearing period, the growth status of each crayfish in every tank was monitored and recorded before each feeding, and mortality events were documented. The daily feeding amount was 5% of the crayfish’s body weight, adjusted according to the actual feeding behavior. Feedings occurred twice daily at 08:30 and 18:30, with each feeding consisting of two rounds separated by a 30 min interval. Every morning after feeding, crayfish excrement was promptly removed, and one-third of the water volume in the tanks was exchanged. The breeding trial lasted for 56 days.

After the official aquaculture period had concluded, a 7-day challenge experiment with *A. veronii* was conducted immediately. Thirty red claw crayfish were arbitrarily chosen from each group (with 10 red claw crayfish selected from each aquaculture tank), resulting in a total of 120 red claw crayfish. Previous experiments had established that the median lethal concentration (LC50) of *A. veronii* infecting red claw crayfish was 1.5 × 10^7^ CFU/mL. The crayfish were then injected with a PBS suspension of *A. veronii* at this LC_50_ concentration into the second abdominal limb segment, with an injection volume of 0.1 mL. Following the injection, the challenged crayfish were returned to their designated rearing tanks according to the pre-established groups and cultured for 7 days, during which they were provided with a basic diet. Mortality was documented daily, and the cumulative mortality was then determined. The calculation of the mortality was based on the following formula:

$$Cumulative\;mortality=\frac{Number\;of\;dead\;crayfish}{Initial\;number\;of\;crayfish}\times 100\%$$

Simultaneously with the cumulative mortality experiment, 60 red claw crayfish were randomly selected from each group (20 per aquaculture tank), resulting in a total of 240 crayfish. A uniform concentration of *A. veronii* (1.5 × 10^7^ CFU/mL) was injected into the second abdominal limb segment of each crayfish. At 0 h, 24 h, 48 h, 72 h, and 96 h post-injection, three red claw crayfish were sampled from each tank using the method described in Section 2.3.

### 2.3. Sampling

A 1 mL disposable syringe was used to withdraw 300 μL of pre-cooled anticoagulant, which was then injected into the hemocoel at the base of the crayfish’s first abdominal segment for hemolymph collection. After needle withdrawal, the collected hemolymph was carefully transferred to a 1.5 mL Eppendorf tube. The sample was centrifuged at 1000× *g* for 10 min at 4 °C. The resulting supernatant was then carefully aspirated and transferred to a fresh Eppendorf tube for storage at −80 °C. Subsequently, the crayfish were transferred to a clean bench. Under sterile conditions, the red claw crayfish were carefully dissected, and the hepatopancreas was excised, immediately frozen in liquid nitrogen, and stored at −80 °C.

### 2.4. Determination of Biochemical Parameters and Antioxidant Enzyme Activities

To measure the biochemical parameters and antioxidant enzyme activities in the hepatopancreas of red claw crayfish, the samples were first thawed. They were rinsed with sterile saline and dried using filter paper. Next, 1 g of hepatopancreas tissue was precisely weighed and transferred into a 50 mL sterile, enzyme-free centrifuge tube. Nine volumes of physiological saline were added to the tube. The sample was then mechanically homogenized on ice and centrifuged at 1000 g for 10 min at 4 °C. The resulting supernatant was collected and diluted with physiological saline to prepare a 1% homogenate for analysis. The biochemical indices of hemolymph were directly detected using the hemolymph supernatant stored at −80 °C after centrifugation.

The contents of total protein (TP) and lysozyme (LZM) and the activities of alkaline phosphatase (AKP), aspartate aminotransferase (AST), and alanine aminotransferase (ALT) in the hemolymph, as well as the activities of AKP, acid phosphatase (ACP), superoxide dismutase (SOD), and catalase (CAT) in the hepatopancreas, were measured using an ELISA analyzer (RT-6100, Rayto, Shenzhen, China) and assay kits. The measurements followed the instructions provided by Nanjing Jiancheng Bioengineering Institute (Nanjing, China). Detailed protocols can be accessed at http://www.njjcbio.com/ (accessed on 8 March 2025).

The TP content in the hemolymph was quantified using the BCA microplate method. The LZM content in the hemolymph was measured by turbidimetry. The AKP activity in the hemolymph was assessed via a micro-enzyme-linked immunosorbent assay. One unit of AKP activity was defined as the amount of enzyme that produces 1 mg of phenol from the substrate in 15 min at 37 °C, using 100 mL of hemolymph, equivalent to 1 King unit per gram of protein (King unit/gprot). The activities of AST (U/L) and ALT (U/L) in the hemolymph were also determined using the microplate method. The activities of AKP and ACP in the hepatopancreas were measured using a micro-enzyme-linked immunosorbent assay. The enzyme activities are expressed in King units per gram of protein (King unit/gprot). One King unit of AKP is defined as the amount of enzyme that produces 1 mg of phenol from the substrate in 15 min at 37 °C, while one King unit of ACP is defined as the amount of enzyme that produces 1 mg of phenol from the substrate in 30 min at 37 °C. The activity of SOD in the hepatopancreas was measured using the WST-1 method. One unit of SOD activity (U/mgprot) was defined as the amount of enzyme required to achieve 50% inhibition of superoxide radicals in a 1 mL reaction mixture containing 1 mg of tissue protein. The CAT activity in the hepatopancreas was determined by the ammonium molybdate method. One unit of CAT activity (U/mgprot) was defined as the amount of enzyme that decomposes 1 μmol of H_2_O_2_ per second per milligram of tissue protein.

### 2.5. Determination of Relative Expression Levels of Genes

According to the methods of Zhang et al. [16], the real-time quantitative polymerase chain reaction (RT-qPCR) was employed to determine the relative expression levels of serine protease inhibitor (*spi*), crustacean hyperglycemic hormone (*chh*), anti-lipopolysaccharide factor (*alf*), superoxide dismutase (*sod*), and *β-actin* in the hepatopancreas of red claw crayfish. *β-actin* was selected as the non-regulated internal reference gene. The forward and reverse primers for RT-qPCR were designed using Primer Premier 6.0 software, based on the mRNA sequences of red claw crayfish retrieved from the NCBI database. The synthesis of these primers was carried out by Shanghai Sangon Bioengineering Technology Co., Ltd. (Shanghai, China). For more detailed information regarding the primers, please refer to Table 2.

The brief steps of the RT-qPCR method are as follows: the hepatopancreas tissue of red claw crayfish was homogenized with sterile physiological saline at a 1:10 (*w/v*) ratio and centrifuged at 1000× *g* for 10 min at 4 °C to obtain the supernatant for RNA extraction. Total RNA was extracted using the Takara MiniBEST Universal RNA Extraction Kit (Takara Biomedical Technology, Beijing, China), with the concentration and purity measured by a NanoDrop-2000 spectrophotometer (Thermo Fisher Scientific, Waltham, Massachusetts, USA). RNA samples meeting the quality criteria (30–1000 ng/μL concentration and A260/A280 ratio of 1.9–2.1) were subjected to integrity verification through 1% agarose TAE gel electrophoresis using GelRed™ nucleic acid stain (UVP, Upland, CA, USA), where intact RNA displayed three distinct bands (28S, 18S, and 5S) with the 28S band intensity being more than twice that of 18S. cDNA was synthesized from 1000 ng total RNA using PrimeScript™ RT Master Mix (Takara) in a 20 μL reaction system containing 10 μL 2× Taqman PCR mix, 1 μL each of forward and reverse primers, RNA template, and DEPC-treated ddH_2_O, under the following thermal conditions: 30 °C for 10 min, 42 °C for 15 min, 95 °C for 5 min, and 5 °C for 5 min. RT-qPCR was performed using the Q2000B Real-Time PCR System (LongGene) with TB Green^®^ Premix Ex Taq™ II (Tli RNaseH Plus, Takara) in a 20 μL reaction volume containing 2 μL cDNA, 10 μL 2× TB Green Premix, 6.4 μL DEPC water, and 0.8 μL each of forward and reverse primers. The RT-qPCR protocol consisted of initial denaturation at 95 °C for 30 s, followed by 40 cycles of 95 °C for 5 s and 60 °C for 20 s. All procedures were carried out under RNase-free conditions to ensure RNA integrity throughout the experimental process.

### 2.6. Data Calculation and Statistics Analysis

In this study, all data were initially organized and processed using Microsoft Excel 2016. Subsequently, one-way ANOVA was performed using SPSS Statistics 25.0 to analyze the experimental results, followed by the generation of relevant graphical representations. Data normality was assessed using a normality test. For analyses involving two distinct categorical independent variables, two-way analysis of variance (ANOVA) or two-way repeated measures ANOVA was applied, followed by Tukey’s post hoc test or Bonferroni’s correction for multiple comparisons to determine significant differences among group means. The Shapiro–Wilk test was used to confirm the normality of the data distribution, while Levene’s test was employed to evaluate the homogeneity of variances. To identify significant intergroup differences, the least significant difference (LSD) test was utilized. GraphPad Prism 9 was utilized for generating the charts. The results of the significance tests are presented as “mean ± standard deviation” (mean ± SD). Different letters denote statistically significant differences (*p* < 0.05) among groups. Additionally, the relative expression levels of the *spi*, *chh*, *alf*, and *sod* genes from fluorescence quantification analysis were calculated using the 2^−ΔΔCT^ method [17].

## 3. Results

### 3.1. Effects of Rhodotorula mucilaginosa on the Cumulative Mortality of Red Claw Crayfish Infected with Aeromonas veronii

The homogeneity of variance test (Brown–Forsythe test) indicated that the assumption of the homogeneity of variance across groups was satisfied (F = 0.604, *p* = 0.921), fulfilling a key prerequisite for analysis of variance. Two-factor ANOVA revealed that both the *R. mucilaginosa* concentrations (F = 23.254, *p* < 0.001, η^2^ = 0.522) and treatment durations (F = 78.71, *p* < 0.001, η^2^ = 0.896) had highly significant main effects on the cumulative mortality rate of red clawed crayfish. However, the interaction between these two factors was not statistically significant (F = 1.147, *p* = 0.328). The model demonstrated strong explanatory power (R^2^ = 0.866), indicating that variations in the mortality rate were primarily independently influenced by *R. mucilaginosa* concentrations and treatment durations, as shown in Table 3 and Table 4.

**Figure 1:**
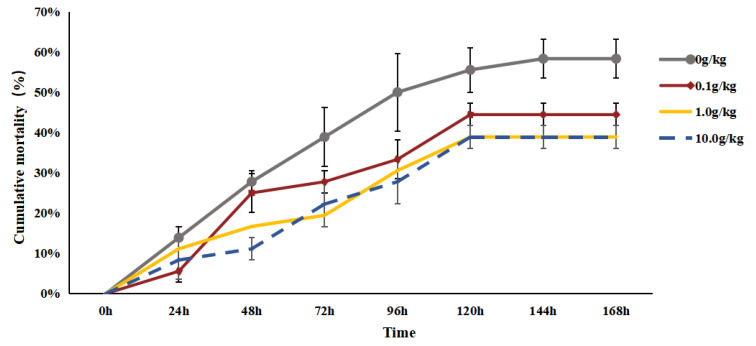
The effects of *Rhodotorula mucilaginosa* on the cumulative mortality of red claw crayfish infected with *Aeromonas veronii*. All the data are presented as the mean ± SD (*n* = 3).

After the injection of *A. veronii*, the mortality rate of the red clawed crayfish in each group kept increasing. Specifically, mortality ceased at 120 h post-injection for the 0.1 g/kg, 1.0 g/kg, and 10.0 g/kg treatment groups, whereas it stopped at 144 h for the control group. Throughout the challenge test period, the cumulative mortalities of the 0.1 g/kg, 1.0 g/kg, and 10.0 g/kg treatment groups were 44.44%, 38.89%, and 38.89%, respectively, which are significantly lower than that of the control group (58.33%) (*p* < 0.05), as shown in Figure 1.

### 3.2. Effects of Rhodotorula mucilaginosa on the Antioxidant and Immune Enzyme Activities in the Hemolymph and Hepatopancreas of Red Claw Crayfish Infected with Aeromonas veronii

The homogeneity of variance test (Brown–Forsythe test) indicated that the assumption of the homogeneity of variance across groups was satisfied (F = 0.517, *p* = 0.891), fulfilling a key prerequisite for analysis of variance. Two-factor ANOVA revealed that both the *R. mucilaginosa* concentrations (F = 104.497, *p* < 0.001, η^2^ = 0.887) and treatment durations (F = 47.097, *p* < 0.001, η^2^ = 0.825) had highly significant main effects on the ACP activity of red clawed crayfish. However, the interaction between these two factors was not statistically significant (F = 1.281, *p* = 0.267). The model demonstrated strong explanatory power (R^2^ = 0.894), indicating that variations in ACP activity were primarily independently influenced by *R. mucilaginosa* concentrations and treatment durations, as shown in Table 5 and Table 6.

**Figure 2:**
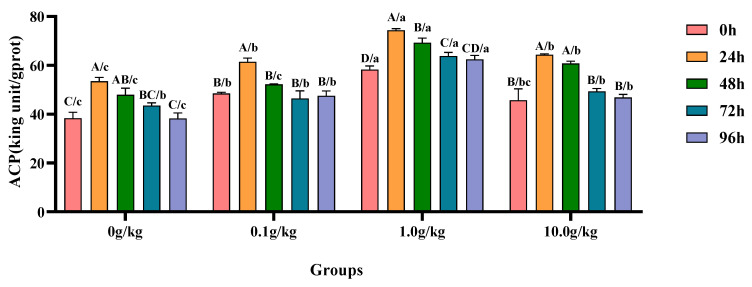
The effects of *Rhodotorula mucilaginosa* on the activity of acid phosphatase (ACP) in the hepatopancreas of red claw crayfish infected with *Aeromonas veronii*. All the data are presented as the mean ± SD (*n* = 3). In the graph, different superscript lowercase letters indicate statistically significant differences (*p* < 0.05) among the values across different groups at the same time. Different superscript capital letters indicate statistically significant differences (*p* < 0.05) among the values across different times within the same group.

Compared with the 0 h time point, infection with *A. veronii* significantly increased the activity of ACP in the hepatopancreas of red claw crayfish at specific time points across different *R. mucilaginosa* dosage groups. In the 0 g/kg and 10.0 g/kg groups, the ACP activity was significantly increased at 24 h and 48 h (*p* < 0.05), with the 0 g/kg group showing 28.4% and 20.1% increases at 24 h and 48 h respectively, while the 10.0 g/kg group exhibited 28.9% and 24.8% increases at the corresponding time points. In the 0.1 g/kg group, the ACP activity significantly increased by 21.0% at 24 h (*p* < 0.05). In the 1.0 g/kg group, the ACP activity showed significant increases of 21.6%, 15.9%, and 8.6% at 24 h, 48 h, and 72 h, respectively (*p* < 0.05). Compared with the 0 g/kg group, after infection with *A. veronii*, the activity of the ACP in the hepatopancreas of red claw crayfish significantly increased at the 0 h time point in the 0.1 g/kg and 1.0 g/kg groups (*p* < 0.05), with enhancements of 21.0% and 34.3%, respectively. At the 24 h and 96 h time points, the ACP activity significantly increased in the 0.1 g/kg, 1.0 g/kg, and 10.0 g/kg groups (*p* < 0.05), with the 0.1 g/kg group showing 12.9% and 19.5% increases, the 1.0 g/kg group demonstrating 28.0% and 38.7% increases, and the 10.0 g/kg group exhibiting 16.8% and 18.3% increases at these time points, respectively. At the 48 h time point, the ACP activity significantly increased by 30.8% and 21.1% in the 1.0 g/kg and 10.0 g/kg groups, respectively (*p* < 0.05). At the 72 h time point, the ACP activity significantly increased by 31.7% in the 1.0 g/kg group (*p* < 0.05), shown in Figure 2.

The homogeneity of variance test (Brown–Forsythe test) indicated that the assumption of the homogeneity of variance across groups was satisfied (F = 0.584, *p* = 0.872), fulfilling a key prerequisite for analysis of variance. Two-factor ANOVA revealed that *R. mucilaginosa* concentrations (F = 107.574, *p* < 0.001, η^2^ = 0.890), treatment durations (F = 147.687, *p* < 0.001, η^2^ = 0.937), and *R. mucilaginosa* concentrations × treatment durations (F = 4.663, *p* < 0.001, η^2^ = 0.583) had highly significant main effects on the AKP activity of red clawed crayfish. The model demonstrated strong explanatory power (R^2^ = 0.942), indicating that variations in the AKP activity were primarily independently influenced by *R. mucilaginosa* concentrations and treatment durations, shown in Table 7 and Table 8.

**Figure 3:**
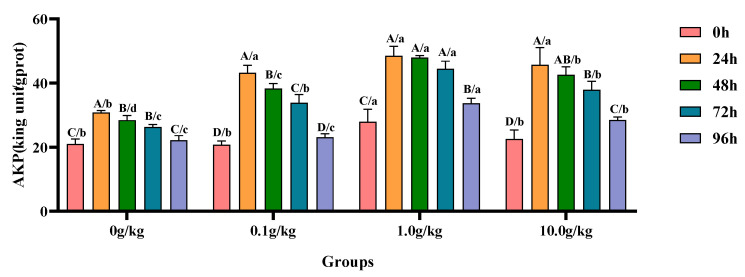
The effects of *Rhodotorula mucilaginosa* on the activity of alkaline phosphatase (AKP) in the hepatopancreas of red claw crayfish infected with *Aeromonas veronii*. All the data are presented as the mean ± SD (*n* = 3). In the graph, different superscript lowercase letters indicate statistically significant differences (*p* < 0.05) among the values across different groups at the same time. Different superscript capital letters indicate statistically significant differences (*p* < 0.05) among the values across different times within the same group.

Compared with the 0 h time point, infection with *A. veronii* significantly increased the activity of AKP in the hepatopancreas of red claw crayfish at specific time points across different *R. mucilaginosa* dosage groups. In the 0 g/kg and 0.1 g/kg groups, the AKP activity significantly increased at 24 h, 48 h, and 72 h (*p* < 0.05), with the 0 g/kg group showing increases of 32.0%, 26.2%, and 20.3%, while the 0.1 g/kg group exhibited increases of 51.9%, 45.8%, and 38.6% at these time points, respectively. In the 1.0 g/kg and 10.0 g/kg groups, the AKP activity significantly increased at 24 h, 48 h, 72 h, and 96 h (*p* < 0.05), with the 1.0 g/kg group demonstrating increases of 42.5%, 41.8%, 37.2%, and 17.2%, while the 10.0 g/kg group showed increases of 50.7%, 47.2%, 40.7%, and 20.9% at these time points, respectively. Compared with the 0 g/kg group, after infection with *A. veronii*, the activity of the AKP in the hepatopancreas of red claw crayfish significantly increased by 24.9% at the 0 h time point in the 1.0 g/kg group (*p* < 0.05). At the 24 h, 48 h, and 72 h time points, the AKP activity significantly increased in the 0.1 g/kg, 1.0 g/kg, and 10.0 g/kg groups (*p* < 0.05), with increases of 28.5%, 36.4%, and 32.5% at 24 h; 25.8%, 40.8%, and 33.3% at 48 h; and 22.2%, 40.8%, and 30.7% at 72 h, respectively. At the 96 h time point, the AKP activity significantly increased by 34.2% and 22.2% in the 1.0 g/kg and 10.0 g/kg groups, respectively (*p* < 0.05), as shown in Figure 3.

The homogeneity of the variance test (Brown–Forsythe test) indicated that the assumption of homogeneity of the variance across groups was satisfied (F = 0.448, *p* = 0.949), fulfilling a key prerequisite for the analysis of variance. Two-factor ANOVA revealed that both the *R. mucilaginosa* concentrations (F = 38.065, *p* < 0.001, η^2^ = 0.741) and treatment durations (F = 70.594, *p* < 0.001, η^2^ = 0.876) had highly significant main effects on the CAT activity of red clawed crayfish. However, the interaction between these two factors was not statistically significant (F = 0.995, *p* = 0.471). The model demonstrated strong explanatory power (R^2^ = 0.868), indicating that the variations in the CAT activity were primarily independently influenced by *R. mucilaginosa* concentrations and treatment durations, as shown in Table 9 and Table 10.

**Figure 4:**
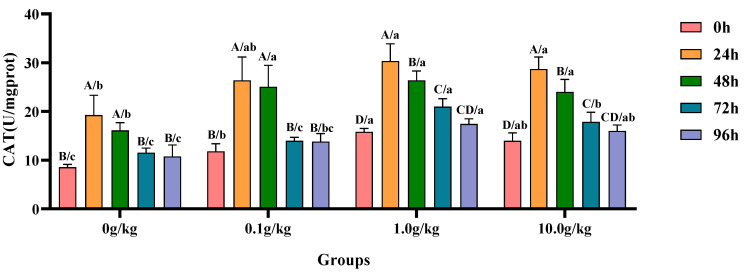
The effects of *Rhodotorula mucilaginosa* on the activity of catalase (CAT) in the hepatopancreas of red claw crayfish infected with *Aeromonas veronii*. All data are presented as the mean ± SD (*n* = 3). In the graph, different superscript lowercase letters indicate statistically significant differences (*p* < 0.05) among the values across the different groups at the same time. Different superscript capital letters indicate statistically significant differences (*p* < 0.05) among the values across different times within the same group.

Compared with the 0 h time point, infection with *A. veronii* significantly increased the activity of CAT in the hepatopancreas of red claw crayfish at specific time points across different *R. mucilaginosa* dosage groups. In the 0 g/kg and 0.1 g/kg groups, CAT activity was significantly increased at 24 h and 48 h (*p* < 0.05), with the 0 g/kg group showing 55.6% and 46.9% increases and the 0.1 g/kg group exhibiting 55.2% and 52.9% increases at these time points, respectively. In the 1.0 g/kg and 10.0 g/kg groups, the CAT activity significantly increased at 24 h, 48 h, and 72 h (*p* < 0.05), with the 1.0 g/kg group demonstrating increases of 47.8%, 40.0%, and 24.6%, while the 10.0 g/kg group showed increases of 51.3%, 41.8%, and 21.8% at these time points, respectively. Compared with the 0 g/kg group, after infection with *A. veronii*, the activity of CAT in the hepatopancreas of red claw crayfish significantly increased at the 0 h and 48 h time points in the 0.1 g/kg, 1.0 g/kg, and 10.0 g/kg groups (*p* < 0.05), with increases of 27.6%, 45.9%, and 38.7% at 0 h and 35.7%, 38.9%, and 32.8% at 48 h respectively. At the 24 h, 72 h, and 96 h time points, the CAT activity significantly increased in the 1.0 g/kg and 10.0 g/kg groups (*p* < 0.05), with increases of 36.5% and 32.9% at 24 h, 45.1% and 35.5% at 72 h, and 38.3% and 32.8% at 96 h, respectively, as shown in Figure 4.

The homogeneity of variance test (Brown–Forsythe test) indicated that the assumption of homogeneity of variance across groups was satisfied (F = 1.042, *p* = 0.482), fulfilling a key prerequisite for the analysis of variance. Two-factor ANOVA revealed that both the *R. mucilaginosa* concentrations (F = 48.854, *p* < 0.001, η^2^ = 0.786) and treatment durations (F = 368.024, *p* < 0.001, η^2^ = 0.974) had highly significant main effects on the SOD activity of red clawed crayfish. The interaction between these two factors was significant (F = 2.536, *p* = 0.014, η^2^ = 0.432). The model demonstrated strong explanatory power (R^2^ = 0.965), indicating that the variations in SOD activity were primarily independently influenced by *R. mucilaginosa* concentrations and treatment durations, as shown in Table 11 and Table 12.

**Figure 5:**
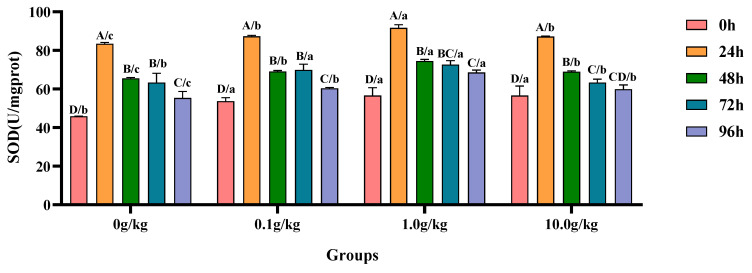
The effects of *Rhodotorula mucilaginosa* on the activity of superoxide dismutase (SOD) in the hepatopancreas of red claw crayfish infected with *Aeromonas veronii*. All data are presented as the mean ± SD (*n* = 3). In the graph, different superscript lowercase letters indicate statistically significant differences (*p* < 0.05) among the values across different groups at the same time. Different superscript capital letters indicate statistically significant differences (*p* < 0.05) among the values across different times within the same group.

Compared with the 0 h time point, infection with *A. veronii* significantly increased the activity of SOD in the hepatopancreas of red claw crayfish at specific time points across different *R. mucilaginosa* dosage groups. In the 0 g/kg, 0.1 g/kg, and 1.0 g/kg groups, the SOD activity significantly increased at 24 h, 48 h, 72 h, and 96 h (*p* < 0.05), with the 0 g/kg group showing increases of 45.1%, 30.0%, 27.7%, and 17.3%, the 0.1 g/kg group exhibiting increases of 38.5%, 22.3%, 23.2%, and 11.0%, and the 1.0 g/kg group demonstrating increases of 38.2%, 24.0%, 22.0%, and 17.4% at these time points, respectively. In the 10.0 g/kg group, the SOD activity was significantly increased at 24 h, 48 h, and 72 h (*p* < 0.05), with increases of 35.1%, 18.0%, and 10.6%, respectively. Compared with the 0 g/kg group, after infection with *A. veronii*, the activity of SOD in the hepatopancreas of red claw crayfish significantly increased at the 0 h, 24 h, 48 h, and 96 h time points in the 0.1 g/kg, 1.0 g/kg, and 10.0 g/kg groups (*p* < 0.05), with increases of 14.7%, 19.1%, and 19.0% at 0 h; 4.3%, 8.9%, and 4.2% at 24 h; 5.2%, 12.1%, and 5.1% at 48 h; and 8.2%, 19.3%, and 7.5% at 96 h, respectively. At the 72 h time point, the SOD activity significantly increased by 9.5% and 12.8% in the 0.1 g/kg and 1.0 g/kg groups, respectively (*p* < 0.05), as shown in Figure 5.

The homogeneity of variance test (Brown–Forsythe test) indicated that the assumption of homogeneity of variance across groups was satisfied (F = 0.613, *p* = 0.822), fulfilling a key prerequisite for the analysis of variance. The two-factor ANOVA revealed that *R. mucilaginosa* concentrations (F = 200.358, *p* < 0.001, η^2^ = 0.938), treatment durations (F = 458.341, *p* < 0.001, η^2^ = 0.979), and *R. mucilaginosa* concentrations × treatment durations (F = 10.695, *p* < 0.001, η^2^ = 0.762) had highly significant main effects on the TP content of the red clawed crayfish. The model demonstrated strong explanatory power (R^2^ = 0.977), indicating that variations in the TP content were primarily independently influenced by *R. mucilaginosa* concentrations and treatment durations, as shown in Table 13 and Table 14.

**Figure 6:**
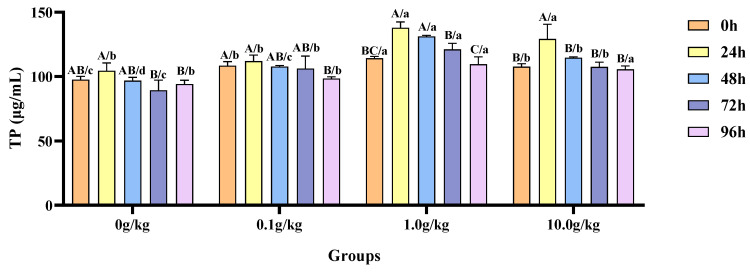
The effects of *Rhodotorula mucilaginosa* on the content of total protein (TP) in the hemolymph of red claw crayfish infected with *Aeromonas veronii*. All data are presented as the mean ± SD (*n* = 3). In the graph, different superscript lowercase letters indicate statistically significant differences (*p* < 0.05) among the values across the different groups at the same time. Different superscript capital letters indicate statistically significant differences (*p* < 0.05) among the values across different times within the same group.

Compared with the 0 h time point, *A. veronii* infection resulted in a significant decrease (10.1%) in hemolymph TP content at 96 h in the 0.1 g/kg group (*p* < 0.05). The 1.0 g/kg group showed significant increases in the TP content of 17.1% at 24 h and 12.9% at 48 h post-infection (*p* < 0.05). In the 10.0 g/kg group, the TP content was significantly elevated by 16.7% at 24 h (*p* < 0.05). The treatment groups exhibited significantly elevated TP content relative to the 0 g/kg control at multiple post-infection time intervals, with the 0.1, 1.0, and 10.0 g/kg groups showing increases of 9.9%, 14.5%, and 9.2% at 0 h, 10.1%, 26.2%, and 15.5% at 48 h and 15.9%, 26.2%, and 17.0% at 72 h, respectively (*p* < 0.05), while the 1.0 and 10.0 g/kg groups maintained significantly higher levels at 24 h (24.2% and 19.2%) and 96 h (14.0% and 10.1%) (*p* < 0.05), as shown in Figure 6.

The homogeneity of variance test (Brown–Forsythe test) indicated that the assumption of homogeneity of variance across groups was satisfied (F = 0.678, *p* = 0.795), fulfilling a key prerequisite for the analysis of variance. Two-factor ANOVA revealed that *R. mucilaginosa* concentrations (F = 2017.012, *p* < 0.001, η^2^ = 0.993), treatment durations (F = 788.309, *p* < 0.001, η^2^ = 0.987), and *R. mucilaginosa* concentrations × treatment durations (F = 90.450, *p* < 0.001, η^2^ = 0.964) had highly significant main effects on the AKP activity of red clawed crayfish. The model demonstrated strong explanatory power (R^2^ = 0.974), indicating that variations in the AKP activity were primarily independently influenced by *R. mucilaginosa* concentrations and treatment durations, shown in Table 15 and Table 16.

**Figure 7:**
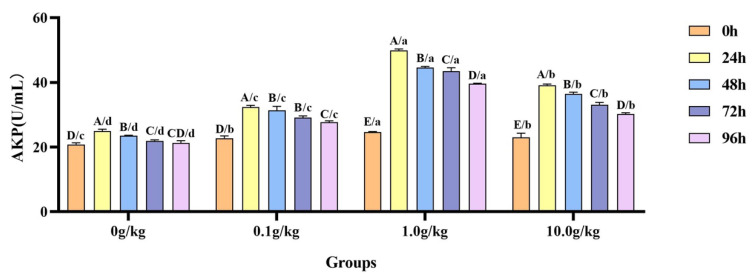
The effects of *Rhodotorula mucilaginosa* on the activity of alkaline phosphatase (AKP) in the hemolymph of red claw crayfish infected with *Aeromonas veronii*. All data are presented as the mean ± SD (*n* = 3). In the graph, different superscript lowercase letters indicate statistically significant differences (*p* < 0.05) among the values across the different groups at the same time. Different superscript capital letters indicate statistically significant differences (*p* < 0.05) among the values across different times within the same group.

A comparative analysis revealed that *A. veronii* infection induced significant temporal increases in hemolymph AKP activity across *R. mucilaginosa* dosage groups (Figure 7). The 0 g/kg group showed 16.4%, 11.5%, and 5.0% elevations at 24 h, 48 h, and 72 h, respectively (*p* < 0.05). More pronounced enhancements were observed in the supplemented groups, as follows: 0.1 g/kg (29.9%, 27.6%, 22.1%, 17.9%), 1.0 g/kg (50.7%, 44.8%, 43.4%, 38.0%), and 10.0 g/kg (41.2%, 36.8%, 30.3%, 24.0%) at corresponding time points (24–96 h) (*p* < 0.05). Compared with the 0 g/kg group, after infection with *A. veronii*, the activity of the AKP in the hemolymph of red claw crayfish significantly increased at the 0 h, 24 h, 48 h, 72 h, and 96 h time points in the 0.1 g/kg, 1.0 g/kg, and 10.0 g/kg groups (*p* < 0.05), with enhancements of 8.3%, 15.4%, and 9.5% at 0 h; 23.1%, 50.1%, and 36.4% at 24 h; 25.1%, 47.2%, and 40.0% at 48 h; 24.8%, 49.6%, and 33.6% at 72 h; and 23.2%, 46.4%, and 29.8% at 96 h, respectively, as shown in Figure 7.

The homogeneity of variance test (Brown–Forsythe test) indicated that the assumption of homogeneity of variance across the groups was satisfied (F = 0.491, *p* = 0.907), fulfilling a key prerequisite for the analysis of variance. Two-factor ANOVA revealed that both the *R. mucilaginosa* concentrations (F = 52.406, *p* < 0.001, η^2^ = 0.979) and treatment durations (F = 155.019, *p* < 0.001, η^2^ = 0.939) had highly significant main effects on the LZM activity of red clawed crayfish. The interaction between these two factors was significant (F = 3.353, *p* = 0.002, η^2^ = 0.501). The model demonstrated strong explanatory power (R^2^ = 0.931), indicating that variations in the LZM activity were primarily independently influenced by *R. mucilaginosa* concentrations and treatment durations, as shown in Table 17 and Table 18.

**Figure 8:**
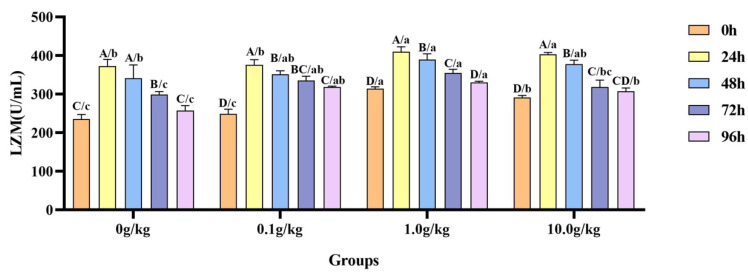
The effects of *Rhodotorula mucilaginosa* on the activity of lysozyme (LZM) in the hemolymph of red claw crayfish infected with *Aeromonas veronii*. All the data are presented as mean ± SD (*n* = 3). In the graph, different superscript lowercase letters indicate statistically significant differences (*p* < 0.05) among the values across different groups at the same time. Different superscript capital letters indicate statistically significant differences (*p* < 0.05) among the values across different times within the same group.

Compared with the 0 h time point, infection with *A. veronii* significantly increased the activity of the LZM in the hemolymph of red claw crayfish at specific time points across different *R. mucilaginosa* dosage groups. In the 0 g/kg, 1.0 g/kg, and 10.0 g/kg groups, the LZM activity significantly increased at 24 h, 48 h, and 72 h (*p* < 0.05), with the activity in the 0 g/kg group being enhanced by 36.8%, 31.0%, and 21.2% at the three time points, the 1.0 g/kg group by 23.4%, 19.4%, and 11.6%, and the 10.0 g/kg group by 27.7%, 22.7%, and 8.3%, respectively. In the 0.1 g/kg group, the LZM activity significantly increased at 24 h, 48 h, 72 h, and 96 h (*p* < 0.05), with elevations of 33.7%, 29.2%, 25.8%, and 21.8%, respectively. Compared with the 0 g/kg group, after infection with *A. veronii*, the activity of the LZM in the hemolymph of red claw crayfish significantly increased at the 0 h and 24 h time points in the 1.0 g/kg and 10.0 g/kg groups (*p* < 0.05), showing enhancements of 25.0% and 19.2% at 0 h and 9.0% and 7.6% at 24 h, respectively. At the 48 h time point, the LZM activity significantly increased by 12.4% in the 1.0 g/kg group (*p* < 0.05). At the 72 h time point, the LZM activity significantly increased in the 0.1 g/kg (10.9%) and 1.0 g/kg (15.8%) groups (*p* < 0.05). At the 96 h time point, the LZM activity was significantly increased in the 0.1 g/kg (19.0%), 1.0 g/kg (21.9%), and 10.0 g/kg (16.1%) groups (*p* < 0.05), as shown in Figure 8.

The homogeneity of variance test (Brown–Forsythe test) indicated that the assumption of homogeneity of variance across the groups was satisfied (F = 0.752, *p* = 0.720), fulfilling a key prerequisite for the analysis of variance. Two-factor ANOVA revealed that both the *R. mucilaginosa* concentrations (F = 53.786, *p* < 0.001, η^2^ = 0.801) and treatment durations (F = 60.722, *p* < 0.001, η^2^ = 0.859) had highly significant main effects on the AST activity of red clawed crayfish. The interaction between these two factors was significant (F = 2.160, *p* = 0.034, η^2^ = 0.393). The model demonstrated strong explanatory power (R^2^ = 0.875), indicating that variations in the AST activity were primarily independently influenced by *R. mucilaginosa* concentrations and treatment durations, as shown in Table 19 and Table 20.

**Figure 9:**
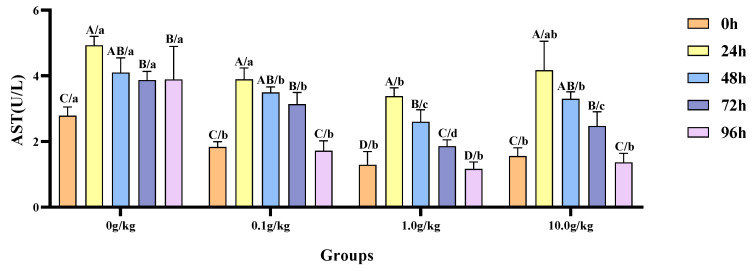
The effects of *Rhodotorula mucilaginosa* on the activity of aspartate aminotransferase (AST) in the hemolymph of red claw crayfish infected with *Aeromonas veronii*. All data are presented as the mean ± SD (*n* = 3). In the graph, different superscript lowercase letters indicate statistically significant differences (*p* < 0.05) among the values across different groups at the same time. Different superscript capital letters indicate statistically significant differences (*p* < 0.05) among the values across different times within the same group.

Relative to the baseline measurements at 0 h, the hemolymph AST activity in red claw crayfish exhibited significant elevation at designated intervals following *A. veronii* infection, with variations observed among distinct dosage cohorts of *R. mucilaginosa* supplementation. Within the 0 g/kg group, enzymatic activity demonstrated statistically substantial rises (*p* < 0.05) of 43.5%, 32.0%, 28.0%, and 28.4% at 24 h, 48 h, 72 h, and 96 h post-infection respectively. The experimental groups receiving 0.1 g/kg, 1.0 g/kg, and 10.0 g/kg dosages displayed marked AST activation (*p* < 0.05) during the 24 h, 48 h, and 72 h examinations, with the 0.1 g/kg cohort showing increments of 53.0%, 47.6%, and 41.7% and the 1.0 g/kg group demonstrating 61.9%, 50.5%, and 30.7% enhancements; while the 10.0 g/kg supplementation yielded 60.2%, 52.8%, and 37.0% augmented activity at corresponding timepoints. When benchmarked against the untreated control, *A.-veronii*-challenged crayfish administered with 0.1 g/kg, 1.0 g/kg, or 10.0 g/kg *R. mucilaginosa* manifested statistically significant suppression (*p* < 0.05) of hemolymph AST levels at 0 h (52.3%, 116.3%, and 79.0% reduction, respectively), 48 h (17.4%, 57.6%, and 24.3%), 72 h (23.3%, 108.0%, and 56.4%), and 96 h (126.4%, 232.8%, and 185.1%), indicating dose-dependent modulation of enzymatic response. Notably, a singular 45.7% diminution (*p* < 0.05) was recorded exclusively in the 1.0 g/kg treatment group at 24 h, as visually documented in Figure 9.

The homogeneity of variance test (Brown–Forsythe test) indicated that the assumption of the homogeneity of variance across groups was satisfied (F = 0.718, *p* = 0.765), fulfilling a key prerequisite for analysis of variance. Two-factor ANOVA revealed that both the *R. mucilaginosa* concentrations (F = 69.984, *p* < 0.001, η^2^ = 0.840) and treatment durations (F = 32.677, *p* < 0.001, η^2^ = 0.766) had highly significant main effects on the ALT activity of red clawed crayfish. However, the interaction between these two factors was not statistically significant (F = 1.933, *p* = 0.059). The model demonstrated strong explanatory power (R^2^ = 0.854), indicating that variations in the ALT activity were primarily independently influenced by *R. mucilaginosa* concentrations and treatment durations, as shown in Table 21 and Table 22.

**Figure 10:**
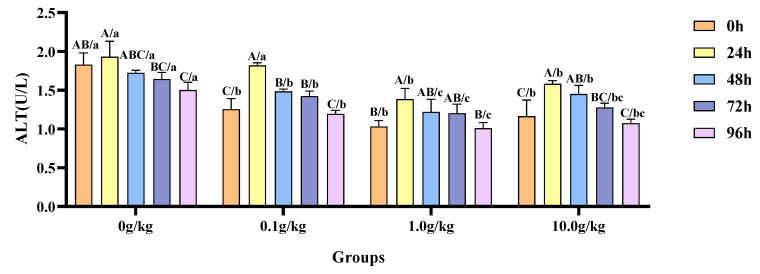
The effects of *Rhodotorula mucilaginosa* on the activity of alanine aminotransferase (ALT) in the hemolymph of red claw crayfish infected with *Aeromonas veronii*. All data are presented as the mean ± SD (*n* = 3). In the graph, different superscript lowercase letters indicate statistically significant differences (*p* < 0.05) among the values across the different groups at the same time. Different superscript capital letters indicate statistically significant differences (*p* < 0.05) among the values across different times within the same group.

Relative to the baseline measurements at 0 h, *A. veronii* infection induced a statistically significant reduction (*p* < 0.05) in hemolymph ALT activity by 21.8% at 96 h in the 0 g/kg group. Conversely, the 0.1 g/kg *R. mucilaginosa* supplementation group exhibited marked declines (*p* < 0.05) in the enzymatic activity—31.0%, 15.5%, and 11.8%—at 24 h, 48 h, and 72 h post-infection, respectively. In the 1.0 g/kg group, the ALT activity was significantly increased by 25.5% at 24 h (*p* < 0.05). The highest dosage group (10.0 g/kg) demonstrated significant attenuation (*p* < 0.05) of ALT activity, with reductions of 26.3% and 19.6% recorded at 24 h and 48 h, respectively. When benchmarked against the untreated control, *A.-veronii*-infected crayfish receiving *R. mucilaginosa* exhibited dose-dependent suppression (*p* < 0.05) of ALT activity at 0 h (45.8%, 77.4%, and 56.9% reductions for 0.1 g/kg, 1.0 g/kg, and 10.0 g/kg groups), 48 h (16.0%, 41.3%, and 18.9%), 72 h (15.4%, 36.6%, and 28.6%), and 96 h (28.5%, 48.6%, and 39.8%), underscoring the probiotic’s regulatory effects across multiple temporal phases. Notably, at 24 h post-infection, the 1.0 g/kg and 10.0 g/kg groups displayed further ALT inhibition (*p* < 0.05) by 39.5% and 22.1%, respectively, as illustrated in Figure 10.

### 3.3. Effects of Rhodotorula mucilaginosa on the Relative Expression Levels of Antioxidant- and Immune-Related Genes in the Hepatopancreas of Red Claw Crayfish Infected with Aeromonas veronii

The homogeneity of variance test (Brown–Forsythe test) indicated that the assumption of homogeneity of variance across the groups was satisfied (F = 1.326, *p* = 0.329), fulfilling a key prerequisite for the analysis of variance. Two-factor ANOVA revealed that *R. mucilaginosa* concentrations (F = 153.178, *p* < 0.001, η^2^ = 0.920), treatment durations (F = 36.672, *p* < 0.001, η^2^ = 0.986), and *R. mucilaginosa* concentrations × treatment durations (F = 6.184, *p* < 0.001, η^2^ = 0.650) had highly significant main effects on the *spi* gene expression of red clawed crayfish. The model demonstrated strong explanatory power (R^2^ = 0.918), indicating that variations in *spi* gene expression were primarily independently influenced by *R. mucilaginosa* concentrations and treatment durations, as shown in Table 23 and Table 24.

**Figure 11:**
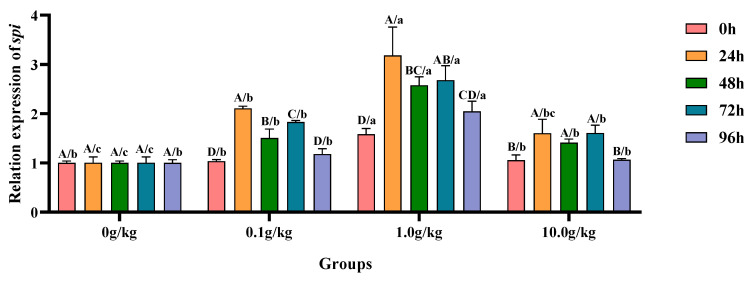
The effects of *Rhodotorula mucilaginosa* on the relative expression levels of the serine protease inhibitor (*spi*) gene in the hepatopancreas of red claw crayfish infected with *Aeromonas veronii*. All the data are presented as mean ± SD (*n* = 3). In the graph, different superscript lowercase letters indicate statistically significant differences (*p* < 0.05) among the values across the different groups at the same time. Different superscript capital letters indicate statistically significant differences (*p* < 0.05) among the values across different times within the same group.

Relative to the baseline levels at 0 h, *A. veronii* challenge induced statistically significant upregulation (*p* < 0.05) of hepatopancreatic *spi* gene expression across all supplemented groups (0.1, 1.0, and 10.0 g/kg *R. mucilaginosa*), with the 0.1 g/kg cohort exhibiting increases of 50.2%, 38.6%, and 40.9% and the 1.0 g/kg group demonstrating 50.9%, 31.2%, and 43.5% elevations; while the 10.0 g/kg group showed more moderate upregulations of 34.2%, 25.2%, and 34.3% at 24 h, 48 h, and 72 h, respectively. When compared with untreated controls, the 1.0 g/kg supplementation group displayed particularly pronounced transcriptional activation (*p* < 0.05) of the *spi* gene following *A. veronii* infection, with expression levels augmented by 36.8% and 51.1% at the initial (0 h) and terminal (96 h) observation periods, respectively. The 24 h post-infection interval revealed striking upregulation (*p* < 0.05) in both the 0.1 g/kg (52.4% increase) and 1.0 g/kg (68.4% increase) treatment groups, indicating particularly robust early-phase genetic responses in these cohorts. Subsequent examinations at 48 h and 72 h documented sustained transcriptional enhancement (*p* < 0.05) across all experimental groups, with 48 h measurements showing 33.6%, 61.2%, and 29.1% increases for 0.1, 1.0, and 10.0 g/kg doses, respectively, while 72 h analyses revealed further upregulation of 45.2%, 62.5%, and 37.5% in corresponding treatment groups, as visually summarized in Figure 11.

The homogeneity of variance test (Brown–Forsythe test) indicated that the assumption of homogeneity of variance across the groups was satisfied (F = 0.645, *p* = 0.825), fulfilling a key prerequisite for analysis of variance. Two-factor ANOVA revealed that both the *R. mucilaginosa* concentrations (F = 63.661, *p* < 0.001, η^2^ = 0.827) and treatment durations (F = 24.067, *p* < 0.001, η^2^ = 0.706) had highly significant main effects on the *sod* gene expression of red clawed crayfish. The interaction between these two factors was significant (F = 3.380, *p* = 0.002, η^2^ = 0.504). The model demonstrated strong explanatory power (R^2^ = 0.840), indicating that variations in the *sod* gene expression were primarily independently influenced by *R. mucilaginosa* concentrations and treatment durations, as shown in Table 25 and Table 26.

**Figure 12:**
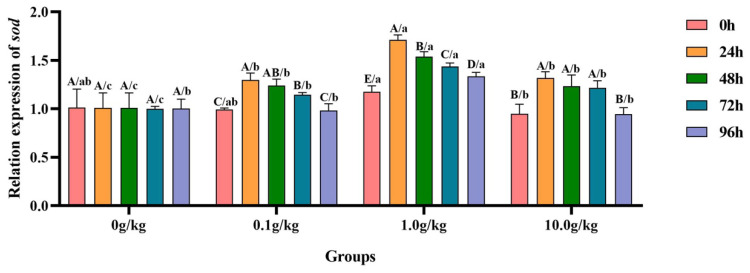
The effects of *Rhodotorula mucilaginosa* on the relative expression levels of the superoxide dismutase (*sod*) gene in the hepatopancreas of red claw crayfish infected with *Aeromonas veronii*. All data are presented as the mean ± SD (*n* = 3). In the graph, different superscript lowercase letters indicate statistically significant differences (*p* < 0.05) among the values across the different groups at the same time. Different superscript capital letters indicate statistically significant differences (*p* < 0.05) among the values across different times within the same group.

Relative to the baseline measurements at 0 h, *A. veronii* infection induced statistically significant upregulation (*p* < 0.05) of the hepatopancreatic *sod* gene expression in both the 0.1 g/kg (23.6%, 20.0%, and 13.5% increases) and 10.0 g/kg (28.0%, 22.9%, and 21.9% increases) treatment groups at 24 h, 48 h, and 72 h post-infection, respectively, demonstrating dose-dependent transcriptional activation patterns. The 1.0 g/kg supplementation group exhibited prolonged upregulation (*p* < 0.05) of the *sod* expression, with elevations of 31.1% at 24 h, 23.6% at 48 h, 18.3% at 72 h, and 12.1% at 96 h, indicating sustained oxidative stress response throughout the experimental period. When benchmarked against untreated controls, all *R.-mucilaginosa*-supplemented groups showed significant enhancement (*p* < 0.05) in *sod* expression following *A. veronii* challenge, as follows: at 24 h (22.2%, 41.0%, and 23.5% for 0.1, 1.0, and 10.0 g/kg, respectively), 48 h (18.6%, 34.4%, and 18.1%), and 72 h (12.7%, 30.4%, and 17.7%), with the 1.0 g/kg dose consistently eliciting the most pronounced transcriptional response across these timepoints. A late-stage upregulation (*p* < 0.05) of 24.9% was uniquely observed in the 1.0 g/kg group at 96 h, suggesting prolonged antioxidant system activation at this optimal probiotic concentration, as visually documented in Figure 12.

The homogeneity of variance test (Brown–Forsythe test) indicated that the assumption of homogeneity of variance across the groups was satisfied (F = 0.692, *p* = 0.781), fulfilling a key prerequisite for the analysis of variance. Two-factor ANOVA revealed that *R. mucilaginosa* concentrations (F = 76.809, *p* < 0.001, η^2^ = 0.852), treatment durations (F = 82.211, *p* < 0.001, η^2^ = 0.892), and *R. mucilaginosa* concentrations × treatment durations (F = 12.943, *p* < 0.001, η^2^ = 0.795) had highly significant main effects on the *chh* gene expression of red clawed crayfish. The model demonstrated strong explanatory power (R^2^ = 0.922), indicating that variations in the *chh* gene expression were primarily independently influenced by *R. mucilaginosa* concentrations and treatment durations, as shown in Table 27 and Table 28.

**Figure 13:**
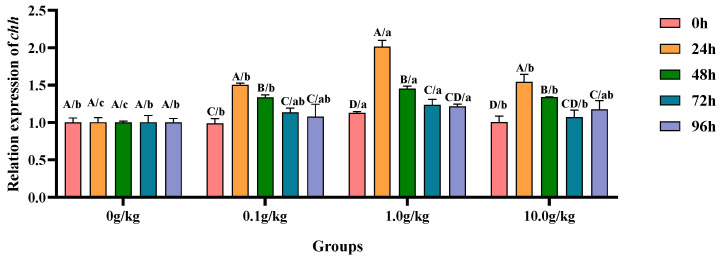
The effects of *Rhodotorula mucilaginosa* on the relative expression levels of the crustacean hyperglycemic hormone (*chh*) gene in the hepatopancreas of red claw crayfish infected with *Aeromonas veronii*. All data are presented as the mean ± SD (*n* = 3). In the graph, different superscript lowercase letters indicate statistically significant differences (*p* < 0.05) among the values across different groups at the same time. Different superscript capital letters indicate statistically significant differences (*p* < 0.05) among the values across different times within the same group.

Relative to the baseline levels at 0 h, *A. veronii* infection induced statistically significant upregulation (*p* < 0.05) of the hepatopancreatic *chh* gene expression in the 0.1 g/kg group, with increases of 34.2% at 24 h and 26.0% at 48 h post-infection, demonstrating early-phase endocrine response modulation. The 1.0 g/kg supplementation cohort exhibited pronounced *chh* transcriptional activation (*p* < 0.05), showing 44.0% elevation at 24 h followed by progressive decreases to 22.5% at 48 h and 8.7% at 72 h, indicating dose-dependent temporal expression patterns. The high-dose (10.0 g/kg) group displayed sustained *chh* upregulation (*p* < 0.05) with 34.9%, 24.9%, and 14.5% increases at 24 h, 72 h, and 96 h, respectively, revealing prolonged endocrine system stimulation at elevated probiotic concentrations. When compared with the untreated controls, the 1.0 g/kg group demonstrated significant *chh* enhancement (*p* < 0.05) at baseline (0 h, 11.2%) and later stages (72 h, 18.9%; 96 h, 17.6%), suggesting this intermediate dosage optimally primes and maintains crustacean hyperglycemic hormone production. Comparative analysis revealed consistent *chh* upregulation (*p* < 0.05) across all supplemented groups during peak response periods, as follows: at 24 h (33.3%, 50.3%, and 35.1% for 0.1, 1.0, and 10.0 g/kg, respectively) and 48 h (25.1%, 31.2%, and 25.2%), with the 1.0 g/kg dose eliciting the maximal transcriptional response, as visually documented in Figure 13.

The homogeneity of variance test (Brown–Forsythe test) indicated that the assumption of homogeneity of variance across the groups was satisfied (F = 0.664, *p* = 0.806), fulfilling a key prerequisite for the analysis of variance. Two-factor ANOVA revealed that the *R. mucilaginosa* concentrations (F = 148.873, *p* < 0.001, η^2^ = 0.918), treatment durations (F = 137.888, *p* < 0.001, η^2^ = 0.932), and *R. mucilaginosa* concentrations × treatment durations (F = 24.759, *p* < 0.001, η^2^ = 0.881) had highly significant main effects on the *alf* gene expression of red clawed crayfish. The model demonstrated strong explanatory power (R^2^ = 0.956), indicating that variations in the *alf* gene expression were primarily independently influenced by *R. mucilaginosa* concentrations and treatment durations, as shown in Table 29 and Table 30.

**Figure 14:**
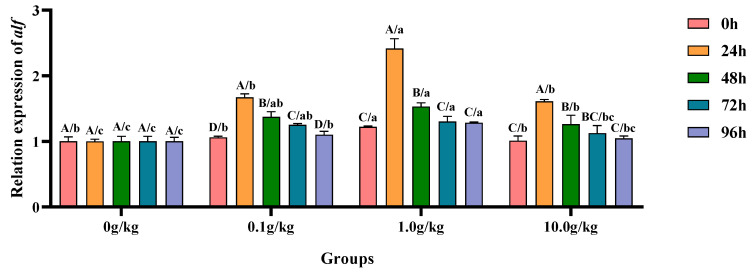
The effects of *Rhodotorula mucilaginosa* on the relative expression levels of the anti-lipopolysaccharide factor (*alf*) gene in the hepatopancreas of red claw crayfish infected with *Aeromonas veronii*. All data are presented as the mean ± SD (*n* = 3). In the graph, different superscript lowercase letters indicate statistically significant differences (*p* < 0.05) among the values across the different groups at the same time. Different superscript capital letters indicate statistically significant differences (*p* < 0.05) among the values across different times within the same group.

Relative to the baseline levels at 0 h, *A. veronii* infection induced significant upregulation (*p* < 0.05) of the hepatopancreatic *alf* gene expression in the 0.1 g/kg group, showing progressive increases of 36.6% at 24 h, 22.9% at 48 h, and 15.3% at 72 h post-infection. Both the 1.0 g/kg and 10.0 g/kg groups exhibited marked *alf* upregulation (*p* < 0.05) during early infection stages, with the 1.0 g/kg group demonstrating 49.5% and 20.3% increases at 24 h and 48 h, respectively, while the 10.0 g/kg group showed 37.2% and 19.9% elevations at the corresponding timepoints. When compared with the untreated controls, the 1.0 g/kg group displayed significant baseline enhancement (*p* < 0.05) of the *alf* expression (17.9% increase at 0 h), suggesting pre-infection priming effects at this optimal dosage. During the peak response periods (24 h and 48 h), all supplemented groups showed significant *alf* activation (*p* < 0.05), as follows: at 24 h (40.1%, 58.6%, and 37.9% for 0.1, 1.0, and 10.0 g/kg, respectively) and 48 h (27.1%, 34.5%, and 20.6%), with the 1.0 g/kg dose consistently eliciting maximal response. Late-phase analyses revealed sustained *alf* upregulation (*p* < 0.05) in the 0.1 g/kg (19.9% at 72 h; 9.0% at 96 h) and 1.0 g/kg (23.1% at 72 h; 22.0% at 96 h) groups, demonstrating prolonged genetic activation particularly at the intermediate dosage, as illustrated in Figure 14.

## 4. Discussion

Infectious diseases are the main obstacle to shrimp culture, and infectious diseases caused by Aeromonas cause significant economic losses to the intensive culture of shrimp [18]. This research showed that *R. mucilaginosa* greatly enhanced the survival rate of red claw crayfish after infection with *A. veronii*. The enhanced host defense may be attributed to the following two synergistic mechanisms: First, immunostimulation via β-glucans, which directly bind to and activate macrophages, neutrophils, and natural killer (NK) cells, thereby potentiating both cellular and humoral immunity [19]. Second, astaxanthin, a type of ketocarotenoid, provides antioxidant defense by acting as a reducing ketone. It stabilizes reactive oxygen species, such as hydrogen peroxide, hydroxyl radicals, and superoxide anions, by transforming these radicals into stable compounds. In doing so, it effectively halts the chain reactions initiated by free radicals [20]. The finding was consistent with previous study on giant freshwater prawns (*Macrobrachium rosenbergii*), where the addition of *Lactobacillus plantarum* improved survival rates when faced with an *Aeromonas hydrophila* challenge, collectively underscoring the efficacy of microbial-derived immunostimulants in aquaculture [21].

Adding probiotics to the feed can enhance the body’s resistance to pathogenic bacteria, and detecting immune indicators is an important means to evaluate the host’s immune ability [22]. In this study, it was found that after the red claw crayfish added with *R. mucilaginosa* were infected by *A. veronii*, the activities of AKP and LZM in the hemolymph and the activities of SOD, CAT, AKP, and ACP in the hepatopancreas increased rapidly in a short amount of time and reached the highest point at 24 h, which is significantly different from the control group. Then, the enzyme activities gradually returned to normal levels, indicating that *R. mucilaginosa* can quickly mobilize the secretion and synthesis of immune and antioxidant enzymes in the body of red claw crayfish when facing the attack of *A. veronii* and improve the body’s ability to respond to pathogen attacks. The dynamic changes in these immune indices, which first increase and then decrease, reflect the complex immune regulatory mechanisms during the interaction between the organism and pathogens. In the early stage of *A. veronii* infection, pathogen-associated molecular patterns (PAMPs) are recognized by pattern recognition receptors (PRRs) in red claw crayfish, rapidly activating the innate immune system [23], and the activities of AKP, ACP, and LZM increase rapidly. Meanwhile, the activities of antioxidant enzymes such as SOD and CAT in the hepatopancreas are significantly enhanced, which can effectively scavenge excessive reactive oxygen species (ROS) induced by pathogen infection and alleviate oxidative stress damage. This is consistent with the mechanism that the organism activates the antioxidant defense system by upregulating the activity of key enzymes (SOD, CAT, GPx, and GST) [24]. As the immune response progresses, the immune regulatory mechanism in red claw crayfish initiates negative feedback regulation to avoid damage to its own tissues caused by excessive immune responses [25]. On the one hand, the excessive production of ROS and immune regulatory factors triggers intracellular antioxidant and anti-inflammatory signaling pathways, inhibiting the continuous expression of immune enzymes and antioxidant enzymes [26]; on the other hand, with the gradual elimination of *A. veronii* by the organism, the immune pressure decreases, causing the activity of related enzymes to gradually return to normal levels, and the organism re-enters immune homeostasis [27].

This study further demonstrated that during the same experimental period, the *R. mucilaginosa* groups exhibited significantly elevated activities of key immune-related enzymes—including hepatopancreatic ACP, AKP, CAT, and SOD, as well as hemolymph AKP and LMZ—compared with the control group, with the 1.0 g/kg group showing particularly pronounced enhancements, indicating that *R. mucilaginosa* effectively augments the innate immune capacity of red claw crayfish through comprehensive modulation of immunoenzymatic systems, an effect likely mediated by bioactive components such as β-glucans and astaxanthin which function dually as pathogen-associated molecular patterns (PAMPs) recognized by host pattern recognition receptors (e.g., Toll-like receptors) to activate downstream innate immune pathways (Toll/IMD) and subsequently upregulate immune enzyme expression [28], while simultaneously serving as immunostimulants that induce controlled reactive oxygen species (ROS) generation leading to compensatory increases in antioxidant enzymes (CAT and SOD) to maintain redox homeostasis, thereby establishing a robust immunophysiological defense network in the crustacean host [29].

This study also revealed that within 24 h following the attack, the activities of ALT and AST in the hemolymph of the four groups of red claw crayfish showed an increase. Subsequently, at the 48 h mark, these activities started to decline, with the enzyme activities slowly returning to normal levels over time. During various stages, the hemolymph of the red claw crayfish in the treatment groups supplemented with *R. mucilaginosa* showed lower ALT and AST activities compared with the control group. This may be because pathogen infection disrupts the normal tissue structure and function of the hepatopancreas. When the hepatopancreas is damaged, intracellular ALT and AST leak in large quantities into the hemolymph, thereby causing the activity of these two enzymes in the hemolymph to increase [30]. *R. mucilaginosa* may reduce the degree of hepatopancreas damage and alleviate the stress level in red claw crayfish by activating the immune system, thereby decreasing the activity of ALT and AST in the hemolymph [31].

This study revealed a characteristic dynamic pattern in the hemolymph ALT and AST activities of red claw crayfish following *A. veronii* infection, exhibiting an increase peaking at 24 h post-infection followed by gradual decline until returning to baseline levels by 96 h, reflecting the typical physiological stress response of crustaceans to pathogenic challenge where early-stage infection (24 h) induces hepatopancreatic tissue damage leading to elevated ALT/AST activities, while subsequent immunological activation (48–96 h) progressively mitigates tissue damage [32]. Notably, the *R. mucilaginosa*-supplemented groups (particularly the 1.0 g/kg dose) maintained significantly lower ALT/AST activities than controls throughout, attributable to dual protective mechanisms, as follows: (1) yeast-derived β-glucans enhancing hemocyte phagocytic capacity through Toll receptor activation [33], thereby reducing direct hepatopancreatic damage by pathogens, and (2) astaxanthin and other antioxidants alleviating oxidative stress via ROS scavenging [34]. These findings align with Grandiosa et al. [35] report of probiotic-enhanced immunity in *Vibrio-splendidus*-challenged New Zealand black-footed abalone (*Haliotis iris*) and Surawut et al. [36] demonstration that a *Bacillus subtilis*, *B. licheniformis*, and *B. megaterium* consortium ameliorated hepatopancreatic necrosis severity in *Vibrio-parahaemolyticus*-infected whiteleg shrimp by boosting immune responses.

This study further revealed that following the challenge, the expression levels of *alf*, *spi*, and *chh* genes in the hepatopancreas of red claw crayfish, which were supplemented with *R. mucilaginosa* in the treatment groups, exhibited a temporary increase. These levels showed significant differences compared with those in the control group across various time points. This may be because when the red claw crayfish is stimulated by bacteria, it will promote the expression of *alf* to produce antibacterial peptides to resist bacterial infection, inhibit the growth and reproduction of bacteria, reduce the damage of bacteria to the body tissues of the shrimp and reduce the severity of infection, thus increasing the survival rate [37]. The up-regulation of *chh* gene expression may be because the *chh* gene regulates energy metabolism and can provide energy support for the body to respond to infection [25]. The up-regulation of the *spi* gene may be because the shrimp body inhibits the protease activity of pathogenic bacteria to prevent the damage of bacteria to the body tissues of the shrimp, thus increasing the survival rate [25]. The up-regulation of *sod* gene expression in the hepatopancreas of the red claw crayfish may be because the immune response of the red claw crayfish is activated after being infected by *A. veronii*, and the innate immune system is activated to up-regulate the expression of *sod* gene to reduce oxidative damage [38].

This study further demonstrated that *R. mucilaginosa* supplementation significantly potentiated this immune response process, with the 1.0 g/kg group showing marked upregulation in the relative expression levels of immune-related genes *spi*, *chh*, *sod*, and *alf*, likely attributable to the yeast’s capacity to activate immunocytes and stimulate cytokine production along with other immunomodulatory mediators that regulate gene expression, thereby enhancing the host’s pathogen-responsive capacity [39]. The astaxanthin component of the red yeast may provide additional protection against ROS-induced oxidative damage, preventing the downregulation of antioxidant genes [40] while concurrently stimulating the expression of oxidative damage repair-associated genes. These observations align with previous reports on white shrimp (*Penaeus vannamei*) [41] and giant tiger prawn (*Penaeus monodon*) [42]. Wang et al. [43] discovered that *R. mucilaginosa* can enhance the immune enzyme activity in Atlantic salmon (*Salmo salar*) following infection by *Aeromonas salmonicida*, thereby strengthening their immune response. This finding aligns with the outcomes of the current study.

## 5. Conclusions

In summary, regarding resistance against *A. veronii*, *R. mucilaginosa* was able to enhance the antibacterial defenses of red claw crayfish. In this experimental context, the ideal addition level of *R. mucilaginosa* was determined to be 1.0 g/kg. Future research will refine this dosage by incorporating a narrower gradient (e.g., 0.8 g/kg and 1.2 g/kg) to determine the most effective addition amount for practical application. These findings will provide a solid scientific foundation for developing efficient compound probiotic feed specifically tailored for red claw crayfish.

## Figures and Tables

**Table 1:** The composition of the experimental diets for red claw crayfish (g / kg of the dried diet).

Ingredient	*R. mucilaginosa* Levels (g/kg)			
	0	0.1	1.0	10.0
*R. mucilaginosa*	0	0.10	1.00	10.00
Fish meal	485.00	485.00	485.00	485.00
Soybean meal	119.30	119.20	118.30	109.30
Sorghum flour	106.50	106.50	106.50	106.50
Wheat flour	139.00	139.00	139.00	139.00
Corn flour	45.00	45.00	45.00	45.00
Soy lecithin	10.00	10.00	10.00	10.00
Fish oil	15.20	15.20	15.20	15.20
Gelatin	20.00	20.00	20.00	20.00
Calcium carbonate	10.00	10.00	10.00	10.00
Choline chloride	5.00	5.00	5.00	5.00
Mineral premixes ^a^	20.00	20.00	20.00	20.00
Vitamin premixes ^b^	20.00	20.00	20.00	20.00
Vitamin C ^c^	5.00	5.00	5.00	5.00
Proximal composition (% Dry Matter)				
Dry material	92.56	92.56	92.56	92.56
Ash	7.76	7.76	7.76	7.76
Ethereal extract	7.40	7.40	7.40	7.40
Crude protein	35.20	35.20	35.20	35.20
Crude lipid	7.84	7.84	7.84	7.84
Fiber	3.43	3.43	3.43	3.43

^a^ Mineral premixes (mg/kg): KCl, 0.5; MgSO_4_·7H_2_O, 0.5; ZnSO_4_·7H_2_O, 0.09; MnCl_2_·4H_2_O, 0.0234; CuSO_4_·5H_2_O, 0.005; KI, 0.005; CoCl_2_·2H_2_O, 0.0025; Na_2_HPO_4_, 2.37. ^b^ Vitamin premixes (mg/kg): vitamin B_12_, 0.02; vitamin A acetate, 5000 IU; vitamin D_3_, 4000 IU; α-tocopherol acetate, 100 IU; menadione, 5; thiamine HCl, 60; riboflavin, 25; pyridoxine HCl, 50; folic acid, 10; dl-capantothenic acid, 75; nicotinic acid, 5; biotin, 1; inositol, 5. ^c^ Vitamin C was provided as ascorbyl phosphate (stabilized form, 35% active ingredient).

**Table 2:** Primer sequences for RT-qPCR in red claw crayfish.

Gene	Primer Sequence (5′→3′)	Amplicon Size (bp)	Tm (°C)	Gene Bank
*β-Actin* ^1^	F: CGCCTGTCCGCTGGAATAAT	135	60	XM_053800817.1
	R: ACGATGGAAGGGAAGACAGC			
*Alp* ^2^	F: GGACGCTCTACTGGCATTGTTACC	111	60	XM_053798842.1
	R: GTCGTTGATGTCGTCGTCACTCTC			
*Chh* ^3^	F: TCTCCGCTCAGCCCTCTACAATG	91	60	XM_053788088.1
	R: AGTGAACGCCCAGAACACACCG			
*Spi* ^4^	F: TGGTCAACGCAGCTTACTTCAAGG	147	60	XM_053782018.1
	R: TGGAGTGGACTAGCCTGAGATTGG			
*Sod* ^5^	F: TGACCTCGGTGACGGCTGTAAG	92	60	XM_053774464.1
	R: CAGCGTGGCGTTCTAAGTCATAGG			

F: forward primer. R: reverse primer. ^1^ *β-Actin*: non-regulated reference gene. ^2^ *Spi*: serine protease inhibitor. ^3^ *Chh*: crustacean hyperglycemic hormone. ^4^ *Alf*: anti-lipopolysaccharide factor. ^5^ *Sod*: superoxide dismutase.

**Table 3:** The variance homogeneity test of cumulative mortality of red claw crayfish across varying *Rhodotorula mucilaginosa* concentrations and treatment durations.

F	df1	df2	*p*
0.604	31	35.389	0.921

Note: The variance homogeneity test of variance was assessed using the Brown–Forsythe test, which is based on the median and adjusts for degrees of freedom.

**Table 4:** The two-way ANOVA with the Tukey post hoc test of the cumulative mortality of red claw crayfish across varying *Rhodotorula mucilaginosa* concentrations and treatment durations.

Source of Variation	SS	df	MS	F	*p*	η^2^
*R. mucilaginosa* concentrations	0.318	3	0.106	23.254	0.000	0.522
Treatment durations	2.511	7	0.359	78.71	0.000	0.896
*R. mucilaginosa* concentrations × Treatment durations	0.11	21	0.005	1.147	0.328	0.273
Error	0.292	64	0.005			
Total variation	3.23	95				

Note: R^2^ = 0.866.

**Table 5:** The variance homogeneity test of acid phosphatase (ACP) activity in the hepatopancreas of red claw crayfish across varying *Rhodotorula mucilaginosa* concentrations and treatment durations.

F	df1	df2	*p*
0.517	19	8.702	0.891

Note: The variance homogeneity test of variance was assessed using the Brown–Forsythe test, which is based on the median and adjusts for degrees of freedom.

**Table 6:** The two-way ANOVA with Tukey post hoc test of the acid phosphatase (ACP) activity in the hepatopancreas of red claw crayfish across varying *Rhodotorula mucilaginosa* concentrations and treatment durations.

Source of Variation	SS	df	MS	F	*p*	η^2^
*R. mucilaginosa* concentrations	3544.284	3	1181.428	104.497	0.000	0.887
Treatment durations	2129.905	4	532.476	47.097	0.000	0.825
*R. mucilaginosa* concentrations × Treatment durations	173.763	12	14.48	1.281	0.267	0.278
Error	452.233	40	11.306			
Total variation	6300.186	59				

Note: R^2^ = 0.894.

**Table 7:** The variance homogeneity test of alkaline phosphatase (AKP) activity in the hepatopancreas of red claw crayfish across varying *Rhodotorula mucilaginosa* concentrations and treatment durations.

F	df1	df2	*p*
0.584	19	17.607	0.872

Note: The variance homogeneity test of variance was assessed using the Brown–Forsythe test, which is based on the median and adjusts for degrees of freedom.

**Table 8:** The two-way ANOVA with Tukey post hoc test of alkaline phosphatase (AKP) activity in the hepatopancreas of red claw crayfish across varying *Rhodotorula mucilaginosa* concentrations and treatment durations.

Source of Variation	SS	df	MS	F	*p*	η^2^
*R. mucilaginosa* concentrations	1736.351	3	578.784	107.574	0.000	0.890
Treatment durations	3178.421	4	794.605	147.687	0.000	0.937
*R. mucilaginosa* concentrations × Treatment durations	301.093	12	25.091	4.663	0.000	0.583
Error	215.213	40	5.38			
Total variation	5431.078	59				

Note: R^2^ = 0.942.

**Table 9:** The variance homogeneity test of catalase (CAT) activity in the hepatopancreas of red claw crayfish across varying *Rhodotorula mucilaginosa* concentrations and treatment durations.

F	df1	df2	*p*
0.448	19	14.631	0.949

Note: The variance homogeneity test of the variance was assessed using the Brown–Forsythe test, which is based on the median and adjusted for degrees of freedom.

**Table 10:** The two-way ANOVA with the Tukey post hoc test of the catalase (CAT) activity in the hepatopancreas of red claw crayfish across varying *Rhodotorula mucilaginosa* concentrations and treatment durations.

Source of Variation	SS	df	MS	F	*p*	η^2^
*R. mucilaginosa* concentrations	656.93	3	218.977	38.065	0.000	0.741
Treatment durations	1624.426	4	406.106	70.594	0.000	0.876
*R. mucilaginosa* concentrations × Treatment durations	68.654	12	5.721	0.995	0.471	0.230
Error	230.108	40	5.753			
Total variation	2580.118	59				

Note: R^2^ = 0.868.

**Table 11:** The variance homogeneity test of superoxide dismutase (SOD) activity in the hepatopancreas of red claw crayfish across varying *Rhodotorula mucilaginosa* concentrations and treatment durations.

F	df1	df2	*p*
1.042	19	12.841	0.482

Note: The variance homogeneity test of variance was assessed using the Brown–Forsythe test, which is based on the median and adjusted for degrees of freedom.

**Table 12:** The two-way ANOVA with Tukey post hoc test of superoxide dismutase (SOD) activity in the hepatopancreas of red claw crayfish across varying *Rhodotorula mucilaginosa* concentrations and treatment durations.

Source of Variation	SS	df	MS	F	*p*	η^2^
*R. mucilaginosa* concentrations	772.335	3	257.445	48.854	0.000	0.786
Treatment durations	7757.466	4	1939.366	368.024	0.000	0.974
*R. mucilaginosa* concentrations × Treatment durations	160.361	12	13.363	2.536	0.014	0.432
Error	210.787	40	5.27			
Total variation	8900.949	59				

Note: R^2^ = 0.965.

**Table 13:** The variance homogeneity test of the total protein (TP) content in the hemolymph of red claw crayfish across varying *Rhodotorula mucilaginosa* concentrations and treatment durations.

F	df1	df2	*p*
0.613	19	8.601	0.822

Note: The variance homogeneity test of variance was assessed using the Brown–Forsythe test, which is based on the median and adjusted for degrees of freedom.

**Table 14:** The two-way ANOVA with Tukey post hoc test of the total protein (TP) content in the hemolymph of red claw crayfish across varying *Rhodotorula mucilaginosa* concentrations and treatment durations.

Source of Variation	SS	df	MS	F	*p*	η^2^
*R. mucilaginosa* concentrations	4654.966	3	1551.655	200.358	0.000	0.938
Treatment durations	14,198.318	4	3549.58	458.341	0.000	0.979
*R. mucilaginosa* concentrations × Treatment durations	993.954	12	82.83	10.695	0.000	0.762
Error	309.776	40	7.744			
Total variation	20,157.014	59				

Note: R^2^ = 0.977.

**Table 15:** The variance homogeneity test of the alkaline phosphatase (AKP) activity in the hemolymph of red claw crayfish across varying *Rhodotorula mucilaginosa* concentrations and treatment durations.

F	df1	df2	*p*
0.678	19	17.86	0.795

Note: The variance homogeneity test of variance was assessed using the Brown–Forsythe test, which is based on the median and adjusted for degrees of freedom.

**Table 16:** The two-way ANOVA with Tukey post hoc test of the alkaline phosphatase (AKP) activity in the hemolymph of red claw crayfish across varying *Rhodotorula mucilaginosa* concentrations and treatment durations.

Source of Variation	SS	df	MS	F	*p*	η^2^
*R. mucilaginosa* concentrations	2533.887	3	844.629	2017.012	0.000	0.993
Treatment durations	1320.426	4	330.106	788.309	0.000	0.987
*R. mucilaginosa* concentrations × Treatment durations	454.514	12	37.876	90.450	0.000	0.964
Error	16.75	40	0.419			
Total variation	4325.577	59				

Note: R^2^ = 0.974.

**Table 17:** The variance homogeneity test of the lysozyme (LZM) activity in the hemolymph of red claw crayfish across varying *Rhodotorula mucilaginosa* concentrations and treatment durations.

F	df1	df2	*p*
0.491	19	8.647	0.907

Note: The variance homogeneity test of variance was assessed using the Brown–Forsythe test, which is based on the median and adjusted for degrees of freedom.

**Table 18:** The two-way ANOVA with Tukey post hoc test of the lysozyme (LZM) activity in the hemolymph of red claw crayfish across varying *Rhodotorula mucilaginosa* concentrations and treatment durations.

Source of Variation	SS	df	MS	F	*p*	η^2^
*R. mucilaginosa* concentrations	27,047.632	3	9015.877	52.406	0.000	0.797
Treatment durations	106,676.994	4	26,669.248	155.019	0.000	0.939
*R. mucilaginosa* concentrations × Treatment durations	6922.779	12	576.898	3.353	0.002	0.501
Error	6881.524	40	172.038			
Total variation	147,528.929	59				

Note: R^2^ = 0.931.

**Table 19:** The variance homogeneity test of the aspartate aminotransferase (AST) activity in the hemolymph of red claw crayfish across varying *Rhodotorula mucilaginosa* concentrations and treatment durations.

F	df1	df2	*p*
0.752	19	12.197	0.720

Note: The variance homogeneity test of variance was assessed using the Brown–Forsythe test, which is based on the median and adjusted for degrees of freedom.

**Table 20:** The two-way ANOVA with Tukey post hoc test of the aspartate aminotransferase (AST) activity in the hemolymph of red claw crayfish across varying *Rhodotorula mucilaginosa* concentrations and treatment durations.

Source of Variation	SS	df	MS	F	*p*	η^2^
*R. mucilaginosa* concentrations	27.524	3	9.175	53.786	0.000	0.801
Treatment durations	41.432	4	10.358	60.722	0.000	0.859
*R. mucilaginosa* concentrations × Treatment durations	4.422	12	0.369	2.16	0.034	0.393
Error	6.823	40	0.171			
Total variation	80.201	59				

Note: R^2^ = 0.875.

**Table 21:** The variance homogeneity test of the alanine aminotransferase (ALT) activity in the hemolymph of red claw crayfish across varying *Rhodotorula mucilaginosa* concentrations and treatment durations.

F	df1	df2	*p*
0.718	19	22.02	0.765

Note: The variance homogeneity test of variance was assessed using the Brown–Forsythe test, which is based on the median and adjusted for degrees of freedom.

**Table 22:** The two-way ANOVA with Tukey post hoc test of alanine aminotransferase (ALT) activity in the hemolymph of red claw crayfish across varying *Rhodotorula mucilaginosa* concentrations and treatment durations.

Source of Variation	SS	df	MS	F	*p*	η^2^
*R. mucilaginosa* concentrations	2.521	3	0.84	69.984	0.000	0.840
Treatment durations	1.57	4	0.392	32.677	0.000	0.766
*R. mucilaginosa* concentrations × Treatment durations	0.278	12	0.023	1.933	0.059	0.367
Error	0.48	40	0.012			
Total variation	4.849	59				

Note: R^2^ = 0.854.

**Table 23:** The variance homogeneity test of relative expression levels of the serine protease inhibitor (*spi*) gene in the hepatopancreas of red claw crayfish across varying *Rhodotorula mucilaginosa* concentrations and treatment durations.

F	df1	df2	*p*
1.326	19	10.232	0.329

Note: The variance homogeneity test of variance was assessed using the Brown–Forsythe test, which is based on the median and adjusted for degrees of freedom.

**Table 24:** The two-way ANOVA with Tukey post hoc test of the relative expression levels of serine protease inhibitor (*spi*) gene in the hepatopancreas of red claw crayfish across varying *Rhodotorula mucilaginosa* concentrations and treatment durations.

Source of Variation	SS	df	MS	F	*p*	η^2^
*R. mucilaginosa* concentrations	16.277	3	5.426	153.178	0.000	0.920
Treatment durations	5.196	4	1.299	36.672	0.000	0.786
*R. mucilaginosa* concentrations × Treatment durations	2.628	12	0.219	6.184	0.000	0.650
Error	1.417	40	0.035			
Total variation	25.518	59				

Note: R^2^ = 0.918.

**Table 25:** The variance homogeneity test of the relative expression levels of superoxide dismutase (*sod*) gene in the hepatopancreas of red claw crayfish across varying *Rhodotorula mucilaginosa* concentrations and treatment durations.

F	df1	df2	*p*
0.645	19	18.129	0.825

Note: The variance homogeneity test of variance was assessed using the Brown–Forsythe test, which is based on the median and adjusted for degrees of freedom.

**Table 26:** The two-way ANOVA with Tukey post hoc test of the relative expression levels of the superoxide dismutase (*sod*) gene in the hepatopancreas of red claw crayfish across varying *Rhodotorula mucilaginosa* concentrations and treatment durations.

Source of Variation	SS	df	MS	F	*p*	η^2^
*R. mucilaginosa* concentrations	1.526	3	0.509	63.661	0.000	0.827
Treatment durations	0.769	4	0.192	24.067	0.000	0.706
*R. mucilaginosa* concentrations × Treatment durations	0.324	12	0.027	3.38	0.002	0.504
Error	0.32	40	0.008			
Total variation	2.938	59				

Note: R^2^ = 0.840.

**Table 27:** The variance homogeneity test of the relative expression levels of the crustacean hyperglycemic hormone (*chh*) gene in the hepatopancreas of red claw crayfish across varying *Rhodotorula mucilaginosa* concentrations and treatment durations.

F	df1	df2	*p*
0.692	19	16.559	0.781

Note: The variance homogeneity test of variance was assessed using the Brown–Forsythe test, which is based on the median and adjusted for degrees of freedom.

**Table 28:** The two-way ANOVA with Tukey post hoc test of the relative expression levels of the crustacean hyperglycemic hormone (*chh*) gene in the hepatopancreas of red claw crayfish across varying *Rhodotorula mucilaginosa* concentrations and treatment durations.

Source of Variation	SS	df	MS	F	*p*	η^2^
*R. mucilaginosa* concentrations	1.254	3	0.418	76.809	0.000	0.852
Treatment durations	1.79	4	0.447	82.211	0.000	0.892
*R. mucilaginosa* concentrations × Treatment durations	0.845	12	0.07	12.943	0.000	0.795
Error	0.218	40	0.005			
Total variation	4.107	59				

Note: R^2^ = 0.922.

**Table 29:** The variance homogeneity test of relative expression levels of anti-lipopolysaccharide factor (*alf*) gene in the hepatopancreas of red claw crayfish across varying *Rhodotorula mucilaginosa* concentrations and treatment durations.

F	df1	df2	*p*
0.664	19	16.911	0.806

Note: The variance homogeneity test of variance was assessed using the Brown–Forsythe test, which is based on the median and adjusted for degrees of freedom.

**Table 30:** The two-way ANOVA with Tukey post hoc test of the relative expression levels of the anti-lipopolysaccharide factor (*alf*) gene in the hepatopancreas of red claw crayfish across varying *Rhodotorula mucilaginosa* concentrations and treatment durations.

Source of Variation	SS	df	MS	F	*p*	η^2^
*R. mucilaginosa* concentrations	2.318	3	0.773	148.873	0.000	0.918
Treatment durations	2.863	4	0.716	137.888	0.000	0.932
*R. mucilaginosa* concentrations × Treatment durations	1.542	12	0.129	24.759	0.000	0.881
Error	0.208	40	0.005			
Total variation	6.931	59				

Note: R^2^ = 0.956.

## Data Availability

The data that support the findings of this study are available from the corresponding author(s) upon reasonable request.
